# Supplementary material for: Modelling Skylarks (Alauda arvensis) to Predict Impacts of Changes in Land Management and Policy: Development and Testing of an Agent-Based Model
Source: PLoS One. 2013 Jun 6;8(6):e65803. doi: 10.1371/journal.pone.0065803 (PMC3675089; doi:10.1371/journal.pone.0065803)
Supplement: Supporting Information S4 — The skylark ODdox as a zipped archive. (ZIP) [file pone.0065803.s004.zip › Skylark_ODdox/farm_8cpp.html]

ALMaSS Skylark ODdox: farm.cpp File Reference


ALMaSS Skylark ODdox
 2.0


- Main Page
- Related Pages
- Classes
- Files

- File List
- File Members

Macros |
Functions |
Variables

farm.cpp File Reference

**Farm.cpp This file contains the source for the Farm class**   
More...

`#include <string.h>`  
`#include <algorithm>`  
`#include <vector>`  
`#include <iostream>`  
`#include <fstream>`  
`#include "../BatchALMaSS/ALMaSS_Setup.h"`  
`#include "../Landscape/ls.h"`  
`#include "../Landscape/cropprogs/Carrots.h"`  
`#include "../Landscape/cropprogs/CloverGrassGrazed1.H"`  
`#include "../Landscape/cropprogs/CloverGrassGrazed2.H"`  
`#include "../Landscape/cropprogs/FieldPeas.h"`  
`#include "../Landscape/cropprogs/Fodderbeet.h"`  
`#include "../Landscape/cropprogs/FodderGrass.h"`  
`#include "../Landscape/cropprogs/Maize.h"`  
`#include "../Landscape/cropprogs/MaizeSilage.h"`  
`#include "../Landscape/cropprogs/OMaizeSilage.h"`  
`#include "../Landscape/cropprogs/OBarleyPeaCloverGrass.h"`  
`#include "../Landscape/cropprogs/OSBarleySilage.h"`  
`#include "../Landscape/cropprogs/OCarrots.h"`  
`#include "../Landscape/cropprogs/OCloverGrassGrazed1.h"`  
`#include "../Landscape/cropprogs/OCloverGrassGrazed2.H"`  
`#include "../Landscape/cropprogs/OCloverGrassSilage1.h"`  
`#include "../Landscape/cropprogs/OFirstYearDanger.h"`  
`#include "../Landscape/cropprogs/OGrazingPigs.h"`  
`#include "../Landscape/cropprogs/OPermanentGrassGrazed.h"`  
`#include "../Landscape/cropprogs/OSpringBarleyPigs.h"`  
`#include "../Landscape/cropprogs/OFieldPeas.h"`  
`#include "../Landscape/cropprogs/OFieldPeasSilage.h"`  
`#include "../Landscape/cropprogs/OOats.h"`  
`#include "../Landscape/cropprogs/Oats.H"`  
`#include "../Landscape/cropprogs/OPotatoes.h"`  
`#include "../Landscape/cropprogs/OSpringBarley.h"`  
`#include "../Landscape/cropprogs/OSpringBarleyExt.h"`  
`#include "../Landscape/cropprogs/OWinterBarley.h"`  
`#include "../Landscape/cropprogs/OWinterBarleyExt.h"`  
`#include "../Landscape/cropprogs/OWinterRape.h"`  
`#include "../Landscape/cropprogs/OWinterRye.h"`  
`#include "../Landscape/cropprogs/OWinterWheatUndersown.h"`  
`#include "../Landscape/cropprogs/OWinterWheatUndersownExt.h"`  
`#include "../Landscape/cropprogs/PermanentGrassGrazed.h"`  
`#include "../Landscape/cropprogs/PermanentGrassLowYield.h"`  
`#include "../Landscape/cropprogs/PermanentGrassTussocky.h"`  
`#include "../Landscape/cropprogs/PermanentSetAside.h"`  
`#include "../Landscape/cropprogs/Potatoes.h"`  
`#include "../Landscape/cropprogs/PotatoesIndustry.h"`  
`#include "../Landscape/cropprogs/seedgrass1.h"`  
`#include "../Landscape/cropprogs/seedgrass2.h"`  
`#include "../Landscape/cropprogs/setaside.H"`  
`#include "../Landscape/cropprogs/SpringBarley.h"`  
`#include "../Landscape/cropprogs/SpringBarleyPTreatment.h"`  
`#include "../Landscape/cropprogs/SpringBarleySKManagement.h"`  
`#include "../Landscape/cropprogs/SpringBarleyCloverGrass.h"`  
`#include "../Landscape/cropprogs/SpringBarleySeed.h"`  
`#include "../Landscape/cropprogs/SpringBarleySilage.h"`  
`#include "../Landscape/cropprogs/SpringRape.H"`  
`#include "../Landscape/cropprogs/Triticale.h"`  
`#include "../Landscape/cropprogs/WinterBarley.h"`  
`#include "../Landscape/cropprogs/winterrape.H"`  
`#include "../Landscape/cropprogs/WinterRye.H"`  
`#include "../Landscape/cropprogs/WinterWheat.H"`  
`#include "../Landscape/cropprogs/WWheatPControl.h"`  
`#include "../Landscape/cropprogs/WWheatPTreatment.h"`  
`#include "../Landscape/cropprogs/WWheatPToxicControl.h"`  
`#include "../Landscape/cropprogs/AgroChemIndustryCereal.h"`  
`#include "../Landscape/cropprogs/WinterWheatStrigling.h"`  
`#include "../Landscape/cropprogs/WinterWheatStriglingCulm.h"`  
`#include "../Landscape/cropprogs/WinterWheatStriglingSingle.h"`  
`#include "../Landscape/cropprogs/SpringBarleyCloverGrassStrigling.h"`  
`#include "../Landscape/cropprogs/SpringBarleyStrigling.h"`  
`#include "../Landscape/cropprogs/SpringBarleyStriglingCulm.h"`  
`#include "../Landscape/cropprogs/SpringBarleyStriglingSingle.h"`  
`#include "../Landscape/cropprogs/MaizeStrigling.h"`  
`#include "../Landscape/cropprogs/winterrapestrigling.h"`  
`#include "../Landscape/cropprogs/WinterRyeStrigling.h"`  
`#include "../Landscape/cropprogs/WinterBarleyStrigling.h"`  
`#include "../Landscape/cropprogs/FieldPeasStrigling.h"`  
`#include "../Landscape/cropprogs/SpringBarleyPeaCloverGrassStrigling.h"`  
`#include "../Landscape/cropprogs/YoungForest.h"`  
`#include "../Landscape/map_cfg.h"`

|  |  |
| --- | --- |
| Macros | |
| #define | \_CRT\_SECURE\_NO\_DEPRECATE |
| #define | \_CRT\_SECURE\_NO\_DEPRECATE |
| #define | \_CRTDBG\_MAP\_ALLOC |

|  |  |
| --- | --- |
| Functions | |
| bool | CompPcts (tpct i, tpct j) |
|  | Used for sorting a farmers field size vector. |

|  |  |
| --- | --- |
| Variables | |
| CfgBool | cfg\_organic\_extensive ("FARM\_ORGANIC\_EXTENSIVE", CFG\_CUSTOM, false) |
| Landscape \* | g\_landscape\_p |
| static CfgStr | l\_map\_farmref\_file ("MAP\_FARMREF\_FILE", CFG\_CUSTOM,"farmrefs.txt") |
| static CfgBool | l\_map\_read\_farmfile ("MAP\_READ\_FARMFILE", CFG\_PRIVATE, true) |

---

## Detailed Description

**Farm.cpp This file contains the source for the Farm class**

by Frank Nikolaisen & Chris J. Topping   
Version of June 2003   
All rights reserved.   
  
Doxygen formatted comments in July 2008

---

## Macro Definition Documentation

#define \_CRT\_SECURE\_NO\_DEPRECATE

#define \_CRT\_SECURE\_NO\_DEPRECATE

#define \_CRTDBG\_MAP\_ALLOC

---

## Function Documentation

|  |  |  |  |
| --- | --- | --- | --- |
| bool CompPcts | ( | tpct | *i*, |
|  |  | tpct | *j* |
|  | ) |  |  |

Used for sorting a farmers field size vector.

References tpct::pct.

Referenced by UserDefinedFarm::AssignPermanentCrop().

{ return (i.pct<j.pct); }

---

## Variable Documentation

CfgBool cfg\_organic\_extensive("FARM\_ORGANIC\_EXTENSIVE", CFG\_CUSTOM, false)

Landscape\* g\_landscape\_p

CfgStr l\_map\_farmref\_file("MAP\_FARMREF\_FILE", CFG\_CUSTOM,"farmrefs.txt")

static

Referenced by FarmManager::FarmManager().

CfgBool l\_map\_read\_farmfile("MAP\_READ\_FARMFILE", CFG\_PRIVATE, true)

static

Referenced by FarmManager::FarmManager().


- CJT
- MSVC
- ALMaSS Working Source
- Landscape
- farm.cpp
- Generated on Thu Jan 10 2013 13:15:35 for ALMaSS Skylark ODdox by
   1.8.1.1
